# Neurovascular coupling and cerebral autoregulation in atrial fibrillation

**DOI:** 10.1177/0271678X19870770

**Published:** 2019-08-19

**Authors:** Rehan T Junejo, Igor D Braz, Samuel JE Lucas, Johannes J van Lieshout, Aaron A Phillips, Gregory YH Lip, James P Fisher

**Affiliations:** 1 School of Sport, Exercise & Rehabilitation Sciences, College of Life & Environmental Sciences, University of Birmingham, Birmingham, UK; 2 Liverpool Centre for Cardiovascular Science, Institute of Ageing and Chronic Disease, University of Liverpool, Liverpool, UK; 3 Medical School, University Center of Volta Redonda, Volta Redonda, Brazil; 4 Centre for Human Brain Health, College of Life & Environmental Sciences, University of Birmingham, Birmingham, UK; 5 Department of Internal Medicine, University of Amsterdam, Amsterdam, The Netherlands; 6 Laboratory for Clinical Cardiovascular Physiology, AMC Center for Heart Failure Research, Academic Medical Center, University of Amsterdam, Amsterdam, The Netherlands; 7 MRC/Arthritis Research UK Centre for Musculoskeletal Ageing Research, School of Life Sciences, University of Nottingham Medical School, Queen's Medical Centre, Nottingham, UK; 8 Departments of Physiology, Pharmacology & Clinical Neurosciences, Libin Cardiovascular Institute, Hotchkiss Brain Institute, Cumming School of Medicine, University of Calgary, Alberta, Canada; 9 Department of Physiology, Faculty of Medical & Health Sciences, University of Auckland, Auckland, New Zealand

**Keywords:** Aging, atrial fibrillation, brain, cerebral circulation, hypertension

## Abstract

The risk of cognitive decline and stroke is increased by atrial fibrillation (AF). We sought to determine whether neurovascular coupling and cerebral autoregulation are blunted in people with AF in comparison with age-matched, patients with hypertension and healthy controls. Neurovascular coupling was assessed using five cycles of visual stimulation for 30 s followed by 30 s with both eyes-closed. Cerebral autoregulation was examined using a sit–stand test, and a repeated squat-to-stand (0.1 Hz) manoeuvre with transfer function analysis of mean arterial pressure (MAP; input) and middle cerebral artery mean blood flow velocity (MCA V_m_; output) relationships at 0.1 Hz. Visual stimulation increased posterior cerebral artery conductance, but the magnitude of the response was blunted in patients with AF (18 [8] %; mean [SD]) and hypertension (17 [8] %), in comparison with healthy controls (26 [9] %) (*P* < 0.05). In contrast, transmission of MAP to MCA V_m_ was greater in AF patients compared to hypertension and healthy controls, indicating diminished cerebral autoregulation. We have shown for the first time that AF patients have impaired neurovascular coupling responses to visual stimulation and diminished cerebral autoregulation. Such deficits in cerebrovascular regulation may contribute to the increased risk of cerebral dysfunction in people with AF.

## Introduction

Atrial fibrillation (AF) is associated with substantial risk of mortality and morbidity from stroke and thromboembolism,^1^^–3^ while ischemic strokes caused by AF are often severe and lead to low quality of life and high mortality.^4^ Even in the absence of previous stroke, patients with AF are at heightened risk of cognitive decline, dementia and depression.^5^^–7^ The precise mechanisms by which AF may precipitate such cognitive dysfunction, and causes such severe cerebrovascular events, have not been fully elucidated but may include impairments in cerebrovascular regulation.

The brain has a high metabolic demand and possesses regulatory mechanisms to ensure a close matching of local blood flow to neuronal activation and metabolism (neurovascular coupling) and an adequate cerebral oxygen delivery over a wide range of perfusion pressures to prevent over/under perfusion and consequent risk of haemorrhage or ischemia (cerebral autoregulation).^8^ Impaired neurovascular coupling and cerebral autoregulation have been identified in a number of pathologies, including stroke and neurovascular diseases^9,10^; however, neither neurovascular coupling nor cerebral autoregulation have been examined in AF. Poor cerebral blood flow in AF may lead to vasodilatation of cerebral arterioles thus reducing cerebral vasodilatory reserve.^11^ Indeed, a 13% reduction in hemispheric cerebral blood flow has been documented in individuals with AF aged between 51 and 65 years, in the absence of signs of heart failure or cerebral vascular disease.^12^ Moreover, systemic endothelial dysfunction has also been reported in AF and may impair cerebrovascular regulation. Plasma von Willebrand factor, a biomarker of endothelial damage/dysfunction, is raised in AF and has been related to adverse outcomes.^13^ In addition, stable patients with chronic AF have an impaired conduit (brachial) artery flow-mediated dilatation response in comparison to sinus rhythm controls,^14^ indicative of attenuated endothelial nitric oxide (NO) bioavailability.^15,16^ This is important since endothelium-derived NO is an important local regulator of cerebral blood flow^17^ and some evidence indicates that it is involved in cerebral autoregulation^18,19^ and neurovascular coupling,^20,21^ although this is controversial^22,23^ and remains an active area of exploration.^17^

We have identified a blunted cerebrovascular carbon dioxide reactivity as an important control mechanism of cerebral blood flow in AF patients^24^ and herein sought to extend those observations by determining whether neurovascular coupling and cerebral autoregulation are impaired in AF. These key cerebrovascular regulatory processes were assessed in patients with AF, hypertension and healthy volunteers. Patients with hypertension were also recruited to serve as a ‘disease control’ group with the aim of controlling for the effect of medications and comorbidities.^24,25^ We hypothesized that neurovascular coupling and cerebral autoregulation would be poorer in patients with AF when compared to age-matched healthy controls and patients with hypertension.

## Material and methods

### Ethical approval

This study was approved by the National Research Ethics Service Committee West Midlands (13/WM/0210 and 15/WM/0447) and adhered to the principles of the Declaration of Helsinki (2008). Prospective participants were provided with an information sheet and all procedures were verbally explained in detail. All volunteers provided written informed consent after having the opportunity to ask questions and receive appropriate clarification.

### Participants

Eighty-three people were recruited to participate in this cross sectional, case control study, across three groups: AF patients (*n* = 30), primary hypertensive patients in sinus rhythm (*n* = 29), and healthy control participants (*n* = 24). AF and hypertension patients were recruited from dedicated clinics at City Hospital, Birmingham, United Kingdom, while healthy controls were recruited from the surrounding communities by recruitment advertisements and word of mouth. In addition, GP practices within the National Institute of Health Research Clinical Research Network were also employed to recruit participants to all groups. Individuals with a fast ventricular rate ( > 90 b/min), left ventricular dysfunction, valvular heart disease, previous myocardial infarction, angina, ischemic and haemorrhagic stroke / transient ischemic attack, malignancy, uncontrolled thyroid disorders, insulin-dependent diabetes, secondary hypertension and respiratory, hepatic, renal, connective tissue, inflammatory or neurological diseases were excluded from participation. Also excluded were those using oral nitrates, hormone replacement therapy, smokers and those with a weekly alcohol consumption of > 28 units. Participant characteristics and prescribed medication use are provided in Table 1.

**Table 1.** Participant characteristics.

	HC	AF	HT	*P* value
*n*	24	30	29	
Age (yr)	68 [66, 70]	69 [63, 72]	68 [65, 72]	0.95
Sex (*n* women)	11	9	14	–
Waist/hip (ratio)	0.84 [0.79, 0.90]	0.86 [0.83, 0.93]	0.88 [0.85, 0.92]	0.38
BMI (kg/m^2^)	24.3 [22.9, 25.6]	27.0 [24.4, 29.8]*	26.0 [25.2, 28.5]*	<0.01
Hypertension (*n*)	–	18	29	–
Type II diabetes (*n*)	–	1	4	–
HR (bpm)	60 [56, 65]	62 [58, 74]	62 [57, 68]	0.28
SBP (mmHg)	141 (21)	135 (24)†	153 (17)	0.01
DBP (mmHg)	81 (08)	82 (12)	85 (11)	0.42
MAP (mmHg)	101 (11)	100 (13)	108 (13)	0.047
MCA V_m_ (cm/s)	62 (14)	51 (13)*	54 (11)	0.01
PCA V_m_ (cm/s)	34 (13)	33 (8)	34 (7)	0.42
P_ET_CO_2_ (mmHg)	40.2 (4.6)	38.1 (5.2)^†^	42.2 (4.3)	0.01
CHADS_2_ Score	–	1 [0, 1]	–	–
CHA_2_DS_2_-VASc Score	–	2 [1, 2]	–	–
Medications (*n*)				
β-blockers	–	12	2	–
ACEi	–	7	11	–
ARB	–	2	7	–
Ca^++^ channel blockers	–	7	13	–
Diuretics	–	3	8	–
OACs	–	22	–	–
Low-dose salicylates	–	3	3	–
Digoxin	–	3	–	–
Statins	5	15	12	–
Biguanides	–	1	4	–
DPP-4 inhibitors	–	–	1	–
Sulfonylureas	–	–	2	–
Thiazolidinediones	–	–	1	–

Note: Values are displayed as mean (SD) when normally distributed or median [interquartile range] when non-normally distributed.

HC: healthy control; AF: atrial fibrillation; HT: hypertension; BMI: body mass index; HR: heart rate; SBP: systolic blood pressure; DBP: diastolic blood pressure; MAP: mean arterial pressure; MCA V_m_: mean middle cerebral artery blood flow velocity; PCA V_m_: mean posterior cerebral artery blood flow velocity; P_ET_CO_2_: end-tidal partial pressure of CO_2_; ACEi: angiotensin-converting enzyme inhibitors; ARB: angiotensin receptor blockers; DPP-4: Dipeptidyl peptidase-4; OACs: oral anticoagulants.

* *P* < 0.05 vs. HC; ^†^*P* < 0.05 vs. HT. PCA signals were obtained at baseline in a subgroup of HC (*n* = 12), AF (*n* = 13) and HT (*n* = 16) participants.

AF patients were previously diagnosed with paroxysmal (i.e. episodes are transient and spontaneously resolve) or persistent AF (i.e. each untreated episode lasts longer than seven days), while patients with hypertension were also previously clinically diagnosed (i.e. consistent non-clinical ambulatory blood pressure readings of systolic >140 mmHg and/or diastolic > 90 mmHg). Four healthy participants were found to have raised blood pressure and were referred to a GP but upon follow-up all were confirmed as being normotensive.

### Experimental measures

Baseline brachial arterial pressure was determined non-invasively using an automated oscillometer (M2, Omron, Kyoto, Japan). Continuous beat-by-beat blood pressure was obtained using finger photoplethysmography (Finometer MIDI, Finapres Medical Systems, Amsterdam, The Netherlands) and heart rate (HR) was monitored by a lead II ECG (BioAmp, ADInstruments, Dunedin, New Zealand). Middle cerebral artery mean blood velocity (MCA V_m_) was continuously recorded in all participants, while posterior cerebral artery mean blood velocity (PCA V_m_) was obtained in a sub-set of participants, using a 2 MHz transcranial Doppler probe (Doppler BoxX, DWL, Sipplingen, Germany) fixed at the temporal window with an adjustable headband and ultrasound gel. MCA insonation was attempted on both sides with recordings made from optimal side; the contralateral PCA was then insonated and again data only from optimally insonated vessels were recorded. Vessels were confirmed in accordance with published guidelines.^26^ Minute ventilation was measured using an oronasal mask connected to a heated pneumotach (Hans Rudolph, Kansas City, KS, USA). Continuous breath-by-breath partial pressure of end-tidal carbon dioxide (P_ET_CO_2_) was obtained by a capnograph connected to the mask by a sample line (RespSense, Nonin Medical, Plymouth, MN, USA).

### Study protocol

#### Baseline

Participants refrained from taking medications (excluding anticoagulants) on the morning before the study. Height and weight were measured. After instrumentation, a 10-min baseline was acquired while participants lay supine breathing room air. At least three brachial artery blood pressure readings were obtained during this period.

#### Neurovascular coupling

Following a 2-min baseline period with both eyes closed, five cycles of 30 s with eyes open (visual stimulation – silent reading) followed by 30 s eyes closed were performed. Reading material consisted of general tourist information relating to Birmingham, UK. The neurovascular coupling protocol was undertaken in accordance with published guidelines.^21^

#### Sit–stand

Cerebral autoregulation was assessed by performing a single sit-to-stand. Participants sat comfortably with the legs uncrossed on a chair for 2 min followed by single movement to a standing position, which was maintained for a further 2 min.

#### Squat-to-stand

Following 2-min of standing, participants performed a repeated squat-to-stand manoeuvre for 5-min (0.1 Hz). Squats consisted of bending the knees at ∼90° for 5 s followed by standing straight for a further 5 s. This manoeuvre produces large amplitude oscillations in blood pressure and cerebral blood flow velocity, thus increasing the coherence between these signals and optimizing the transfer function analysis method and its reproducibility.^27,28^

### Data analysis

Study design restricted blinding during data collection. However, researchers were blinded during data and statistical analysis. Body mass index (BMI) was expressed as the ratio between participant's weight and the square of their height. Cardiorespiratory and transcranial Doppler signals were digitized at 1 kHz (Powerlab, ADInstruments) and recorded using multi-channel data acquisition software (LabChart 7, ADInstruments). Mean blood pressure of each cardiac cycle was calculated as the mean arterial pressure (MAP), while an index of the cerebrovascular conductance (CVCi) was calculated using the following formula

$$\text{MC}{\text{A}}_{\text{CVCi}}\;\text{or}\;\text{PC}{\text{A}}_{\text{CVCi}}=\frac{\text{MCA}{\text{V}}_{\text{m}}\;\text{or}\;\text{PCA}{\text{V}}_{\text{m}}}{\text{MAP}}$$

Baseline values for cardiorespiratory and cerebrovascular data were averaged over 10 min. For neurovascular coupling analysis, beat-to-beat (cardiovascular and cerebrovascular) and breath-by-breath (respiratory) data underwent cubic spline interpolation at 5 Hz using a custom built Matlab code (MathWorks, United States). The average values of MCA V_m_, MCA_CVCi_, PCA V_m_, PCA_CVCi_, MAP and P_ET_CO_2_ were calculated for the eyes open and eyes closed phases. Additionally, peak responses to eyes open were computed and expressed as percentage change from eyes closed. The magnitude of the neurovascular coupling response was principally taken as the peak percentage change in PCA_CVCi_. A secondary analysis was undertaken in an attempt to better understand the “task-specific selectivity” of the response, in which the “non-selective” cerebrovascular responses to the neurovascular coupling test were excluded by calculating the difference between the PCA_CVCi_ and MCA_CVCi_ responses.

Cerebral autoregulation was determined from cardiovascular and cerebrovascular responses to the sit–stand manoeuvre, along with the transfer function analysis of cardiovascular (MAP) and cerebrovascular (MCA V_m_) signals for the supine baseline (5 min) and the repeated squat-to-stand manoeuvre (5 min). For sit–stand data, baseline values of MCA V_m_, MCA_CVCi_, MAP and P_ET_CO_2_ were calculated as the average of 20 s data before stand (i.e. while seated). The magnitude of the change (delta; Δ) to nadir was calculated along with the time to nadir. For the repeated squat-to-stand, extracted data underwent cubic polynomial interpolation and resampling at 10 Hz for transfer function analysis using the fast Fourier transform in accordance with the recommendations of the International Cerebral Autoregulation Research Network (CARNet, http://www.car-net.org/content/resources).^29^ The relationship between input (MAP) and output (MCA V_m_) was quantified with respects to power, absolute gain (cm/s/mmHg), normalised gain (%/mmHg), phase and coherence, averaged at 0.1 Hz (i.e. coincident with frequency of repeated squat to stand).^27^ In addition, averages were computed over the very low frequency (0.02–0.07 Hz), low frequency (0.07–0.2 Hz), and high frequency (0.2–0.5 Hz) ranges.^30^

### Statistical analyses

Participant numbers included for each analysis are shown in the figure and table legends. Normality was assessed by the Shapiro–Wilk test. Normally distributed data were analyzed using one-way analysis of variance (ANOVA) or Students *t*-test, while non-normally distributed data were analyzed using Kruskal–Wallis H test or Mann–Whitney Rank Sum test. Multiple pairwise comparisons were performed using Students *t*-test with Bonferroni correction if a significant difference was found by the ANOVA. When a significant difference between the groups was found by the Kruskal–Wallis H test, post hoc analysis was performed with Dunn's method. Statistical analysis was performed using Sigmaplot 13.0 (Systat Software Inc., London, UK). Significance was set at *P* < 0.05. Normally distributed data are presented as mean (SD), while non-normally distributed data are presented as median [interquartile range]. Assuming a 6% SD for NVC as Nowak-Fluck et al.,^31^ the minimal detectable difference between the groups would be 7% with a sample size of 12 patients in each group, at 80% power and 5% alpha. Assuming a 0.35%/mmHg SD for normalized gain at 0.1 Hz during the repeated squat-to-stand manoeuvre as Smirl et al.,^32^ the minimal detectable difference between the groups would be 0.28%/mmHg with a sample size of 25 patients in each group, at 80% power and 5% alpha.

## Results

### Baseline

Groups were closely matched for age and waist/hip ratio, while BMI was higher in AF and hypertension patients (Table 1). During the supine baseline, HR and DBP were not different between groups, while SBP and MAP were lowest in AF and highest in hypertension. Supine MCA V_m_ and P_ET_CO_2_ were lower in AF, while PCA V_m_ was not different between groups.

### Neurovascular coupling

**Figure 1.**
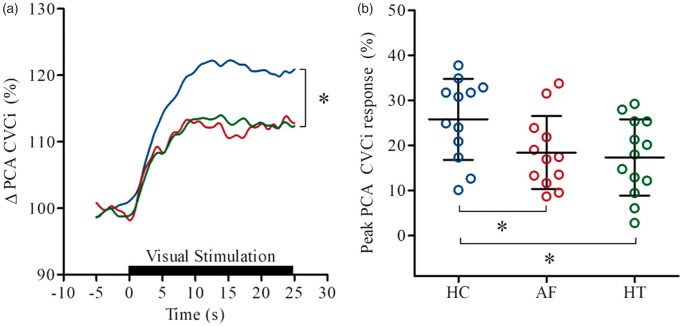
Neurovascular coupling response in the posterior (PCA; a) in healthy controls (HC; blue), patients with atrial fibrillation (AF; red) and hypertension (HT; green). Lines represent the group mean responses. The black bar indicates where participants had their eyes open. Panel b shows the individual peak PCA_CVCi_ responses (%) with mean (SD) for each group. *P < 0.05 vs HC.

Visual stimulation evoked an increase in PCA_CVCi_ in all groups (Figure 1 and Table 2), but the magnitude of the hyperaemic response was blunted in patients with AF and hypertension, in comparison with healthy controls (*P* < 0.05). AF patients exhibited the greatest increase in MCA_CVCi_ (*P* < 0.05 vs. healthy controls and patients with hypertension) and when this “non-selective” effect of the neurovascular coupling test was subtracted, a significantly diminished peak PCA_CVCi_ response was observed in AF patients compared to both other groups. When the peak MCA V_m_ was similarly accounted for, the peak PCA V_m_ was also observed to be diminished in AF patients (*P* < 0.05 vs. healthy controls and patients with hypertension). The neurovascular coupling test slightly elevated P_ET_CO_2_, but to a similar extent in each group. MAP was also slightly elevated in the AF and hypertension patient groups (*P* < 0.01 vs. healthy controls).

**Table 2.** Neurovascular coupling (NVC) responses in healthy controls (HC), patients with atrial fibrillation (AF) and hypertension (HT).

	HC	AF	HT	*P* value
*n*	12	12	13	
Baseline				
PCA V_m_ (cm/s)	36 (8)	31 (7)	33 (8)	0.36
MCA V_m_ (cm/s)	60 (15)	49 (12)	55 (12)	0.13
MAP (mmHg)	99 (10)	106 (13)	112 (14)	0.06
MCA_CVCi_ (cm/s/mmHg)	0.61 (0.17)	0.47 (0.13)	0.50 (0.12)*	<0.05
PCA_CVCi_ (cm/s/mmHg)	0.36 (0.09)	0.30 (0.07)	0.30 (0.07)	0.06
P_ET_CO_2_ (mmHg)	41 [39–43]	39 [36–40]	43 [40–45]	0.07
Peak NVC response				
Δ PCA V_m_ (%)	24 (10)	19 (7)	19 (7)	0.21
Δ MCA V_m_ (%)	11 (7)	19 (6)^*†^	12 (3)	<0.01
Δ PCA V_m_-MCA V_m_ (%)	12.5 (8.1)	0.1 (7.6)^*§^	6.8 (8.7)	<0.01
Δ MAP (%)	1 (2)	4 (3)*	3 (2)*	<0.01
Δ PCA_CVCi_ (%)	26 (9)	18 (8)*	17 (8)*	<0.05
Δ MCA_CVCi_ (%)	13 (6)	17 (6)^†^	10 (4)	<0.05
Δ PCA_CVCi_-MCA_CVCi_ (%)	12.9 (9.2)	1.0 (7.5)*	6.6 (9.4)	<0.01
Δ P_ET_CO_2_ (%)	3 [100–104]	2 [101–104]	2 [100–104]	0.51
Δ PCA V_m_ / P_ET_CO_2_ (%)	23 (8)	19 (8)	18 (8)	0.26
Δ MCA V_m_ / P_ET_CO_2_ (%)	11 (6)	19 (7)^*†^	12 (5)	<0.01

Note: Values are displayed as mean (SD) when normally distributed or median [interquartile range] when non-normally distributed. *P* values represent ANOVA results.

MCA: middle cerebral artery; PCA: posterior cerebral artery; V_m_: mean blood flow velocity; MAP: mean arterial pressure; CVCi: cerebrovascular conductance index; P_ET_CO_2_: end-tidal partial pressure of CO_2_.

*P < 0.05 vs. HC; ^†^*P* < 0.05 vs. HT; ^§^*P* = 0.056 vs. HT.

### Cerebral autoregulation

**Figure 2.**
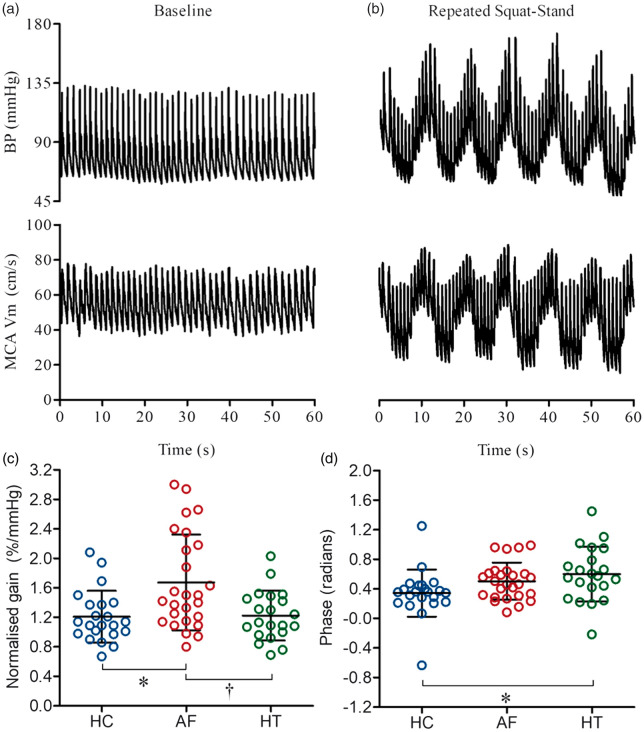
Cerebral autoregulation determined using transfer function analysis during a repeated squat-stand manoeuvre at 0.10 Hz in healthy controls (HC; blue), patients with atrial fibrillation (AF; red) and hypertension (HT; green). Panels a and b show original records of arterial blood pressure (BP) and middle cerebral artery mean blood flow velocity (MCA V_m_) at baseline (supine) and during repeated squat-stands in one healthy participant. Panels c and d show transfer function normalised gain and phase determined during the repeated squat-stand manoeuvre at 0.10 Hz in each study group with mean (SD). *P < 0.05 vs. HC; ^†^*P* < 0.05 vs. HT.

Cardiovascular and cerebrovascular responses to the sit-stand manoeuvre are provided in Table 3. No differences were noted between the groups in terms of the magnitude of the fall in MAP, MCA V_m_ or MCA_CVCi_. Similarly, no between group differences were noted for the time taken to reach a nadir. Figure 2 and Supplementary Table 1 provide transfer function analysis derived coherence, phase and gain in the very low, low and high frequency ranges, along with 0.1 Hz, both at baseline and during a repeated squat-stand manoeuvre. Normalised gain was elevated in patients with AF compared to patients with hypertension during the repeated squat-stand manoeuvre at 0.1 Hz, compared to normotensive controls in sinus rhythm (*P* < 0.05). Coherence and absolute gain were not different between the groups during the repeated squat-stand manoeuvre at 0.1 Hz; however, phase was lower in patients with hypertension when compared to normotensive controls in sinus rhythm (*P* < 0.05). As anticipated, the coherence was appreciably increased by performance of the squat-stand manoeuvre in all groups.

**Table 3.** Cerebrovascular, haemodynamic and respiratory responses to a single sit-stand manoeuvre in healthy controls (HC), patients with atrial fibrillation (AF) and hypertension (HT).

	HC	AF	HT	*P* value
*n*	24	30	29	
Baseline (sitting)				
MCA V_m_ (cm/s)	59 (17)	53 (14)	49 (10)*	0.03
MAP (mmHg)	100 [94–108]	98 [83–112]^†^	117 [108–128]*	<0.01
MCA_CVCi_ (cm/s/mmHg)	0.54 [0.46–0.77]	0.51 [0.39–0.64]^†^	0.42 [0.35–0.46]*	<0.01
P_ET_CO_2_ (mmHg)	37 [35–39]	38 [33–40]	38 [36–40]	0.39
Change from sitting to nadir during standing				
Δ MCA V_m_ (%)	14 [18–9]	15 [17–8]	11 [15–6]	0.23
Time to nadir of MCA V_m_ (s)	23 [6–34]	27 [6–33]	25 [8–32]	0.90
Δ MAP (%)	20 (8)	19 (10)	16 (9)	0.15
Time to nadir of MAP (s)	9 [8–11]	8 [7–11]	10 [8–12]	0.16
Δ MCA_CVCi_ (%)	10 [14–8]	11 [16–3]	7 [13–5]	0.47
Time to nadir of CVCi (s)	27 [22–40]	25 [3–34]	30 [4–40]	0.74
Δ P_ET_CO_2_ (%)	5 [10–3]	7 [9–5]	6 [9–2]	0.85

Note: Values are displayed as mean (SD) when normally distributed or median [interquartile range] when non-normally distributed. *P* values represent ANOVA results. ^*^P < 0.05 vs. HC; ^†^*P* < 0.05 vs. HT.

MCA: middle cerebral artery; V_m_: mean blood flow velocity; MAP: mean arterial pressure; CVCi: cerebrovascular conductance index; P_ET_CO_2_: end-tidal partial pressure of CO_2_.

### Subgroup analysis of AF patients being tested during an AF episode or not

Approximately 50% of AF patients were in AF when studied, and so a subgroup analysis was undertaken between these patients compared to those not in AF when tested. Groups were not different in terms of age, hip-to-waist ratio, SBP, PCA V_m_, P_ET_CO_2_, whereas HR, BMI (tendency, *P* = 0.08), DBP (tendency, *P* = 0.06) and MAP (tendency, *P* = 0.06) were higher, and MCA V_m_ was lower in those AF patients that were fibrillating when tested (Supplementary Table 2). Neither neurovascular coupling (Supplementary Table 3), nor the cardiovascular and cerebrovascular responses to the sit-stand manoeuvre were different in these sub-groups of AF patients (Supplementary Table 4). Supplementary Table 5 provides transfer function analysis derived coherence, phase and gain at 0.1 Hz, both at baseline and during a repeated squat-stand manoeuvre. Interestingly, during the squat-stand manoeuvre, absolute gain was lower (0.64 [0.22] vs. 0.92 [0.37] cm/s/mmHg) and phase was higher (0.63 [0.25] vs. 0.35 [0.17] radians) in AF patients that were fibrillating when studied (*P* < 0.05).

## Discussion

The aim of this study was to determine whether neurovascular coupling and cerebral autoregulation are impaired in AF. Our major novel findings are that: (1) individuals with AF have a blunted neurovascular coupling response to visual stimulation when compared to age-matched healthy controls; (2) cerebral autoregulation is diminished in AF patients as indicated from the elevated normalised gain derived from transfer function analysis, and (3) AF patients that were fibrillating when studied had a lower cerebral perfusion (MCA V_m_) than those AF patients not in AF when tested, but cerebral autoregulatory performance was not less effective.

### Neurovascular coupling

A complex array of signalling cascades normally couples neuronal activation and metabolism to cerebral blood flow with a high spatial and temporal fidelity.^20^ A diminished neurovascular coupling, indicative of cerebrovascular dysfunction, has been demonstrated in several clinical conditions (e.g., stroke, Alzheimer's disease),^9,21^ and while an association between impaired neurovascular coupling and diminished cognitive function has been reported,^33^ human studies of causality are awaited. In the present study, we observed that increases in visual stimulation evoked a blunted PCA_CVCi_ response in patients with AF compared to healthy control participants, indicative of diminished neurovascular coupling. This may be attributable to visual stimulation causing a blunted neuronal activation in AF as sub-clinical neurodegeneration can occur in some cases,^34^ or alternatively a disruption in the coupling between neuronal activation and increases in local blood flow.

A multitude of substances are believed to link neuronal and astrocytic activation to local vasodilatation, including vasoactive ions (e.g. potassium, hydrogen), metabolites (e.g. adenosine, lactate), neurotransmitters (e.g. glutamate, dopamine) and factors derived from the endothelium (e.g. NO).^21,35^ Endothelial damage/dysfunction has been reported in AF, and may in part be due to blood flow abnormalities associated with an irregular heart rate, with resultant turbulent flow both in the left atrium and systemically.^36^ Loss of shear stress, which occurs in conditions of turbulent flow, decreases endothelial NO synthase expression as compared with laminar or pulsatile flow conditions.^37,38^ Diminished NO signalling may contribute to the diminished neurovascular coupling we report.^35^ However, in contrast to the abundance of studies employing isolated preparations and non-human models, the mechanistic basis of neurovascular coupling has been more sparsely studied in humans.^21^ Further studies are needed to better understand the physiological mechanisms for the observed deficit in the cerebral perfusion response of the occipital lobe to visual stimulation in AF patients.

Patients with hypertension were observed to have a lower PCA_CVCi_ response to visual stimulation in comparison with healthy control participants. This is in general agreement with a previous report of a blunted cerebral blood flow response in the parietal cortex during a memory task in untreated hypertension patients compared to normotensive controls.^33^ Interestingly, a poorer cerebral blood flow response was associated with a worse cognitive performance.^33^ In mice, the systemic administration of angiotensin II raises blood pressure and diminishes the cerebral blood flow response to whisker stimulation (i.e. a blunted neurovascular coupling response).^39^ Given that phenylephrine administration to induce a similar pressor response to angiotensin II did not blunt the hyperaemic response to whisker stimulation, hypertension *per se* does not appear to explain the blunted neurovascular coupling and perhaps points to an effect of angiotensin II on a vascular regulation (e.g. vascular oxidative stress, diminished NO bioavailability).^39,40^ In the present study, PCA_CVCi_ responses to visual stimulation were similar in patients with AF and hypertension. This occurred despite the known right-shift in the cerebral autoregulation curve documented in hypertension, which is reported to have a complex influence on the neurovascular coupling response,^41,42^ and may relate to the diminished vasoactive signalling mechanisms described above. Of note, AF patients exhibited a larger MCA_CVCi_ and MCA V_m_ response than patients with hypertension. The MCA does not supply the visual cortex and this “non-selective” response may reflect the engagement of cognitive pathways that are independent of visual cortex stimulation.^43^ Interestingly, when the “non-selective” MCA_CVCi_ and MCA V_m_ responses to the neurovascular coupling test were subtracted from the PCA_CVCi_ and PCA V_m_ responses in a secondary analysis, AF patients displayed the poorest neurovascular coupling of all the groups studied (i.e. the lowest percentage increase in PCA_CVCi_ and PCA V_m_). Diminished neurovascular coupling, may over time, disrupts the neuronal microenvironment and causes functional deficits in the brain.

### Cerebral autoregulation

An impaired cerebral autoregulation has been identified in conditions including traumatic brain injury,^44^ Alzheimer's disease,^45^ type 2 diabetes mellitus^46^ and following mild ischaemic stroke.^47^ In accordance with previous studies, we observed that MCA V_m_ (an index of cerebral perfusion) was reduced in AF patients in the supine position, and this has previously been postulated to be both a cause^12^ and a consequence^11^ of an impaired cerebral autoregulation. A number of complex mechanisms are involved in the autoregulatory process and include stretch-activated cation channels,^48^ autonomic influences^49^ metabolic influences (e.g. CO_2_),^50^ and NO,^18,19^ although the latter is still debated.^17^ As described above, endothelial damage/ dysfunction and diminished endothelial NO bioavailability have been identified in AF,^13,14^ along with autonomic imbalance.^51^ Our study is the first to determine whether cerebral autoregulation is impaired in AF and this was assessed using two approaches. First, the examination of the temporal pattern of the MCA V_m_ and MAP responses to a simple sit–stand manoeuvre, and second, the transfer function analysis of these same signals during a repeated squat-to-stand manoeuvre. Importantly, these manoeuvres have high ecological validity as they commonly occur in normal everyday life (e.g. rising from a chair or standing up after retrieving something from the floor) and can be accompanied by symptoms indicative of cerebral hypoperfusion (e.g. light-headedness). Both manoeuvres evoke pronounced changes in blood pressure, and in the case of the repeated squat-to-stand manoeuvre, the large oscillations in blood pressure evoked are transmitted to cerebral perfusion (Figure 2), thus increasing the coherence between these variables and optimizing the transfer function analysis method and its reproducibility.^27,28^

In line with our hypothesis, cerebral autoregulation was diminished in AF patients compared to either healthy controls or patients with hypertension (i.e. normalised gain was elevated). A higher gain is usually indicative of poorer buffering of changes in MAP by the cerebral vasculature, but it can also suggest a decreased cerebral vascular resistance.^29^ P_ET_CO_2_ was similar between groups during sit–stand test and slightly diminished in AF patients compared to those with hypertension, during supine baseline coincident with transfer function analysis. Of note, as hypercapnia decreases autoregulation,^50^ these differing P_ET_CO_2_ levels are unlikely to explain the group differences observed in cerebral autoregulation. A sub-group analysis of AF patients that were fibrillating (paroxysmal and persistent AF) versus those not in AF (paroxysmal AF) when tested identified that the former group had a reduced transfer function absolute gain and increased phase. Phase is representative of the delay between the input (MAP) and output (MCA V_m_), and a lower phase shift may indicate a more passive following of MCA V_m_ to MAP, thus a reduced cerebral autoregulatory performance.^52,53^ The lower absolute gain and increased phase observed suggest a more effective cerebral autoregulation in the AF patients that were fibrillating when studied. This puzzling observation may be directly attributable to the fibrillation per se, but the contribution of a secondary related factor, such as the higher MAP (tendency) and lower MCA V_m_, cannot be excluded. Indeed, normalised gain, which negates group differences in MCA V_m_, was not different between AF sub-groups. Additional studies are required to better understand the mechanisms, whereby cerebral autoregulation may be altered in this population. Interestingly, there were no between-group differences in the cerebral perfusion response to the single sit–stand manoeuvre, suggesting that the more haemodynamically strenuous manoeuvre (i.e. repeated squat-to-stand) was required to reveal the AF-related alterations in cerebral autoregulation.

### Limitations

The reported changes in MCA V_m_ and PCA V_m_ derived using transcranial Doppler ultrasound are only proportional to cerebral blood flow if the cross-sectional area of the insonated artery remains unchanged. Of note, the transcranial Doppler technique is the only practical way we could have quantified the cerebral perfusion responses to posturally induced changes in blood pressure and thus derive cerebral autoregulatory data. An increase in AF burden from paroxysmal to persistent, and the duration of AF and hypertension since diagnosis, might be expected to further worsen cerebrovascular function. However, this information for individual patients was not obtained thus limiting further within group analysis. Investigations of sub-clinical indices of end-organ damage and cerebral scans for covert infarcts were beyond the scope of the protocol and as such were not performed.

## Conclusions

Neurovascular coupling and cerebral autoregulation are diminished in AF patients compared to age-matched, healthy controls and patients with hypertension. Future studies should explore the molecular basis for these observations, along with their clinical implications as they may be significant in terms of AF-related cognitive decline and brain dysfunction.

## Supplemental Material

Supplemental material for Neurovascular coupling and cerebral autoregulation in atrial fibrillation Click here for additional data file. Supplemental Material for Neurovascular coupling and cerebral autoregulation in atrial fibrillation by Rehan T Junejo, Igor D Braz, Samuel JE Lucas, Johannes J van Lieshout MD Aaron A Phillips, Gregory YH Lip and James P Fisher in Journal of Cerebral Blood Flow & Metabolism
