# Supplementary material for: Neurovascular coupling and cerebral autoregulation in atrial fibrillation
Source: J Cereb Blood Flow Metab. 2019 Aug 19;40(8):1647–57. doi: 10.1177/0271678X19870770 (PMC7370373; doi:10.1177/0271678X19870770)
Supplement: Supplemental material for Neurovascular coupling and cerebral autoregulation in atrial fibrillation [file Supplemental_Material1.pdf]

SUPPLEMENTARY MATERIAL

Supplementary Table I. Transfer function analysis assessment of dynamic cerebral autoregulation at baseline (supine) and during the repeated squat-stand manoeuvres in healthy controls (HC), patients with atrial fibrillation (AF) and hypertension (HT).

Supplementary Table II. Atrial fibrillation patient characteristics.

Supplementary Table III. Neurovascular coupling responses in patients with atrial fibrillation that were not fibrillating or fibrillating when examined**.**

Supplementary Table IV. Cerebrovascular, haemodynamic and respiratory responses to a single sit-stand manoeuvre in patients with atrial fibrillation that were not fibrillating or fibrillating when examined**.**

Supplementary Table V. Transfer function analysis assessment of dynamic cerebral autoregulation at baseline (supine) and during the repeated squat-stand manoeuvres in patients with atrial fibrillation that were not fibrillating or fibrillating when examined**.**

<u>Supplementary Table I</u>. Transfer function analysis assessment of dynamic cerebral autoregulation at baseline (supine) and during the repeated squat-stand manoeuvres in healthy controls (HC), patients with atrial fibrillation (AF) and hypertension (HT).

		**HC**	**AF**	**HT**	**P value**
		VLF (0.02–0.07 Hz)			
MAP power (mmHg^2)	Baseline	5.45 [2.73-8.50]	6.22 [3.46-11.57]	3.76 [2.40-7.42]	0.285
	Squat-stand	6.11 [4.05-10.52]	7.70 [4.23-12.48]	8.40 [4.37-9.72]	0.862
MCA V_m power (cm^2/s^{-2})	Baseline	2.74 [1.35-4.43]	2.05 [1.36-3.15]	1.95 [1.06-3.30]	0.539
	Squat-stand	3.57 [1.69-5.67]	3.90 [1.92-6.17]†	2.05 [0.90-3.70]	0.030
Coherence	Baseline	0.52 (0.20)	0.54 (0.17)	0.45 (0.13)	0.142
	Squat-stand	0.56 (0.17)	0.56 (0.16)	0.51 (0.12)	0.527
Absolute gain (cm/s/mmHg)	Baseline	0.55 [0.45-0.64]	0.47 [0.39-0.71]	0.43 [0.37-0.66]	0.418
	Squat-stand	0.61 [0.42-0.73]	0.51 [0.39-0.73]†	0.39 [0.33-0.45]*	0.005
Normalised gain (%/mmHg)	Baseline	0.96 [0.66-1.15]	1.00 [0.94-1.11]	0.90 [0.71-1.09]	0.284
	Squat-stand	1.02 [0.72-1.15]	1.15 [0.85-1.56]†	0.78 [0.69-0.88]	0.002
Phase (radians)	Baseline	1.04 [0.76-1.18]	1.17 [0.77-1.34]	0.90 [0.59-1.14]	0.142
	Squat-stand	0.74 (0.34)	0.67 (0.31)	0.76 (0.28)	0.597
		LF (0.07–0.20 Hz)			
MAP power (mmHg^2)	Baseline	2.40 [1.82-4.41]	6.59 [2.54-10.55]†	2.45 [1.17-5.54]	0.032
	Squat-stand	128.35 [41.18-203.30]	72.65 [34.41-186.73]	23.42 [15.60-106.77]*	0.011
MCA V_m power (cm^2/s^{-2})	Baseline	1.85 [1.42-3.00]	3.85 [2.22-4.37]†	1.28 [0.84-2.76]	0.006
	Squat-stand	68.74 [19.80-154.95]	47.84 [16.55-94.93]	10.00 [6.37-30.71]*	0.014

Coherence	Baseline	0.77 [0.70-0.83]	0.80 [0.74-0.88]	0.75 [0.55-0.84]	0.265
	Squat-stand	0.75 [0.60-0.85]	0.72 [0.57-0.84]	0.66 [0.55-0.77]	0.450
Absolute gain (cm/s/mmHg)	Baseline	0.83 [0.62-0.92]	0.73 [0.58-0.82]	0.63 [0.55-0.83]	0.213
	Squat-stand	0.69 [0.47-0.85]	0.64 [0.51-0.80]	0.59 [0.43-0.65]	0.250
Normalised gain (%/mmHg)	Baseline	1.21 [1.06-1.47]	1.55 [1.29-1.67]†‡	1.26 [0.98-1.44]	0.015
	Squat-stand	1.05 [0.91-1.23]	1.27 [1.08-1.96]	1.21 [1.01-1.36]	0.062
Phase (radians)	Baseline	0.64 (0.22)	0.55 (0.27)	0.61 (0.24)	0.447
	Squat-stand	0.27 (0.24)	0.36 (0.18)	0.43 (0.30)	0.106
		HF (0.2–0.5 Hz)			
MAP power ($mmHg^2$)	Baseline	0.58 [0.28-1.19]	3.22 [1.86-6.33]*†	0.56 [-0.36-1.27]	<0.001
	Squat-stand	12.51 [7.43-21.15]	20.15 [8.39-29.47]	7.72 [5.09-21.99]	0.098
MCA V_m power (cm^2/s^{-2})	Baseline	0.60 [0.28-1.00]	2.43 [0.94-4.38]*†	0.45 [0.38-0.92]	<0.001
	Squat-stand	5.57 [4.52-9.24]	8.56 [5.66-11.24]†	3.62 [-1.97-5.78]	0.003
Coherence	Baseline	0.58 (0.26)	0.77 (0.18)*†	0.51 (0.26)	<0.001
	Squat-stand	0.63 (0.20)	0.64 (0.18)	0.58 (0.19)	0.593
Absolute gain (cm/s/mmHg)	Baseline	0.89 [0.65-1.19]	0.70 [0.58-0.89]	0.73 [0.59-0.90]	0.166
	Squat-stand	0.61 [0.44-0.73]	0.52 [0.44-0.72]	0.58 [0.45-0.68]	0.651
Normalised gain (%/mmHg)	Baseline	1.15 [1.16-1.85]	1.54 [1.25-1.71]	1.38 [1.17-1.62]	0.405
	Squat-stand	0.96 [0.81-1.12]	1.32 [0.93-1.70]	0.51 [0.48-0.68]	0.076
Phase (radians)	Baseline	0.06 [-0.06-0.15]	0.004 [-0.05-0.08]	0.06 [-0.02-0.21]	0.344
	Squat-stand	-0.06 (0.29)	0.06 (0.21)	-0.02 (0.58)	0.211

		0.1 Hz			
Coherence	Baseline	0.73 [0.65-0.83]	0.83 [0.68-0.89]	0.78 [0.52-0.86]	0.185
	Squat-stand	0.99 [0.97-1.00]	0.98 [0.89-0.99]	0.94 [0.86-0.99]	0.052
Absolute gain (cm/s/mmHg)	Baseline	0.73 (0.24)	0.66 (0.14)	0.56 (0.18)*	0.012
	Squat-stand	0.72 [0.53-0.92]	0.71 [0.59-0.91]	0.54 [0.44-0.80]	0.184
Normalised gain (%/mmHg)	Baseline	1.18 (0.37)	1.35 (0.32)†	1.01 (0.27)	0.002
	Squat-stand	1.12 [0.99-1.37]	1.46 [1.16-2.16]*†	1.13 [1.00-1.45]	0.007
Phase (radians)	Baseline	0.74 [0.62-0.89]	0.72 [0.50-1.01]	0.66 [0.54-0.87]	0.586
	Squat-stand	-0.04 [-0.13-0.04]	0.48 [0.31-0.61]	0.57 [0.42-0.79]*	0.016

Values are displayed as mean (SD) when normally distributed or median [interquartile range] when non-normally distributed. * P < 0.05 vs HC, † P < 0.05 vs HT.

Supplementary Table II. Atrial fibrillation patient characteristics.

	**Non Fibrillating**	**Fibrillating**	**P value**
n	14	16	
Age (yr)	66 (10)	68 (6)	0.45
Sex (n female)	5	4	-
BMI (kg/m^2)	24.7 [22.6, 29.1]	27.8 [25.9, 31.6]	0.08
Waist/Hip (ratio)	0.83 (0.09)	0.87 (0.14)	0.55
$CHADS_2$	0 [0, 2]	1 [0, 1]	0.65
CHA_2DS_2-VASc	2 (1)	2 [1, 2]	0.80
Hypertension (n)	6	12	-
Type II Diabetes (n)	-	1	-
HR (bpm)	59 [54, 61]	69 [62, 76]*	<0.01
SBP (mmHg)	129 (29)	141 (17)	0.17
DBP (mmHg)	78 (13)	86 (10)	0.06
MAP (mmHg)	96 (12)	104 (12)	0.06
MCA V_m (cm/s)	59 (10)	44 (11)*	<0.01
PCA V_m (cm/s)	36 (10)	32 (6)	0.40
$P_{ET}CO_2$ (mmHg)	38.3 (4.4)	38.0 (5.9)	0.90

Values are displayed as mean (SD) when normally distributed or median [interquartile range] when non-normally distributed. BMI, body mass index; HR, heart rate; SBP, systolic blood pressure; DBP, diastolic blood pressure; MAP, mean arterial pressure; MCA, middle cerebral artery; PCA, posterior cerebral artery; V_m, mean blood flow velocity; $P_{ET}CO_2$, partial pressure of end-tidal CO_2. PCA recordings obtained in n=4 non fibrillating and n=9 fibrillating patients. *P < 0.05 vs fibrillating.

<u>Supplementary Table III</u>. Neurovascular coupling responses in patients with atrial fibrillation that were not fibrillating or fibrillating when examined.

	Non Fibrillating	Fibrillating	P value
n	3	9	
	Baseline		
PCA V_m (cm/s)	37 (10)	29 (5)	0.09
MCA V_m (cm/s)	57 (5)	47 (12)	0.21
MAP (mmHg)	104 [95-104]	116 [101-117]	0.46
MCA_{CVCi} (cm/s/mmHg)	0.58 (0.07)	0.44 (0.12)	0.09
PCA_{CVCi} (cm/s/mmHg)	0.36 [0.33-0.41]	0.27 [0.24-0.29]*	0.04
$P_{ET}CO_2$ (mmHg)	35 (6)	39 (3)	0.12
	Peak NVC response		
Δ PCA V_m (%)	19 (7)	20 (7)	0.84
Δ MCA V_m (%)	116 [111-122]	121 [117-124]	0.58
Δ PCA V_m - MCA V_m (%)	2.0 (9.7)	-0.5 (7.3)	0.64
Δ MAP (%)	3 (3)	5 (2)	0.28
Δ PCA_{CVCi} (%)	19 (5)	18 (9)	0.95
Δ MCA_{CVCi} (%)	16 (9)	18 (6)	0.70
Δ PCA_{CVCi}-MCA_{CVCi} (%)	2.6(9.9)	0.5 (7.2)	0.69
Δ $P_{ET}CO_2$ (%)	1 [101-106]	3 [102-104]	0.36
Δ PCA V_m / $P_{ET}CO_2$ (%)	18 (7)	19 (9)	0.88
Δ MCA V_m / $P_{ET}CO_2$ (%)	18 [114-122]	18 [112-128]	1.00

Values are displayed as mean (SD) when normally distributed or median [interquartile range] when non-normally distributed. Abbreviations: MCA, middle cerebral artery; PCA, posterior cerebral artery; V_m, mean blood flow velocity; MAP, mean arterial pressure; CVCi, cerebrovascular conductance index; $P_{ET}CO_2$, end-tidal partial pressure of CO_2. *$P < 0.05$ vs fibrillating.

<u>Supplementary Table IV</u>. Cerebrovascular, haemodynamic and respiratory responses to a single sit-stand manoeuvre in patients with atrial fibrillation that were not fibrillating or fibrillating when examined**.**

	**Non Fibrillating**	**Fibrillating**	**P value**
n	13	17	
Baseline (sitting)			
MCA V_m (cm/s)	58 (13)	48 (13)*	0.04
MAP (mmHg)	93 (19)	106 (23)	0.09
MCA CVCi (cm/s/mmHg)	0.66 (0.21)	0.48 (0.16)*	0.01
$P_{ET}CO_2$ (mmHg)	37 [33-40]	38 [33-39]	0.61
Change from sitting to nadir during standing			
Δ MCA V_m (%)	14 (8)	13 (7)	0.78
Time to nadir of MCA V_m (s)	26 [9-29]	29 [5-33]	0.93
Δ MAP (%)	21 (12)	18 (7)	0.32
Time to nadir of MAP (s)	9 [7-11]	8 [7-10]	0.45
Δ MCA CVCi (%)	7 (10)	11 (10)	0.30
Time to nadir of CVCi (s)	22 [3-35]	28 [3-33]	0.93
Δ $P_{ET}CO_2$ (%)	7 [10-5]	7 [9-4]	0.71

Values are displayed as mean (SD) when normally distributed or median [interquartile range] when non-normally distributed. Abbreviations: MCA, middle cerebral artery; V_m, mean blood flow velocity; MAP, mean arterial pressure; CVCi, cerebrovascular conductance index; $P_{ET}CO_2$, end-tidal partial pressure of CO_2. *$P < 0.05$ vs fibrillating.

Supplementary Table V. Transfer function analysis assessment of dynamic cerebral autoregulation at baseline (supine) and during the repeated squat-stand manoeuvres in patients with atrial fibrillation that were not fibrillating or fibrillating when examined**.**

		**Non Fibrillating**	**Fibrillating**	**P value**
n		12	14	
Coherence	Baseline	0.72 [0.65-0.81]	0.87 [0.82-0.90]*	0.02
	Squat-stand	0.99 [0.98-1.00]	0.94 [0.85-0.98]*	0.04
Absolute gain (cm/s/mmHg)	Baseline	0.73 (0.13)	0.60 (0.12)*	0.02
	Squat-stand	0.92 (0.37)	0.64 (0.22)*	0.02
Normalised gain (%/mmHg)	Baseline	1.29 [1.07-1.35]	1.48 [1.13-1.56]	0.17
	Squat-stand	1.56 [1.30-2.23]	1.39 [1.11-1.80]	0.29
Phase (radians)	Baseline	0.75 [0.38-0.97]	0.72 [0.57-1.04]	0.59
	Squat-stand	0.35 (0.17)	0.63 (0.25)*	<0.01

Values are displayed as mean (SD) when normally distributed or median [interquartile range] when non-normally distributed. *P < 0.05 vs fibrillating.
